# Loss of FYCO1 leads to cataract formation

**DOI:** 10.1038/s41598-021-93110-1

**Published:** 2021-07-02

**Authors:** Kiyotoshi Satoh, Yukitoshi Takemura, Motohiko Satoh, Kiyokazu Ozaki, Shunichiro Kubota

**Affiliations:** 1 Department of Life Sciences, Graduate School of Arts and Sciences, The University of Tokyo, 3-8-1 Komaba, Meguro-ku, Tokyo, 153-8902 Japan; 2 Laboratory of Pathology, Setsunan University, 45-1 Nagaotohge-cho, Hirakata, Osaka Japan

**Keywords:** Autophagy, Organelles, Cell biology, Biochemistry, Proteins

## Abstract

Autophagy is a degradation process of cytoplasmic proteins and organelles trafficked to degradation vesicles known as autophagosomes. The conversion of LC3-I to LC3-II is an essential step of autophagosome formation, and FYCO1 is a LC3-binding protein that mediates autophagosome transport. The p62 protein also directly binds to LC3 and is degraded by autophagy. In the present study, we demonstrated that disrupting the FYCO1 gene in mice resulted in cataract formation. LC3 conversion decreased in eyes from FYCO1 knockout mice. Further, FYCO1 interacted with αA- and αB-crystallin, as demonstrated by yeast two-hybrid screening and immunoprecipitation analyses. In eyes from knockout mice, the soluble forms of αA- and αB-crystallin, the lens’s major protein components, decreased. In addition, p62 accumulated in eyes from FYCO1 knockout mice. Collectively, these findings suggested that FYCO1 recruited damaged α-crystallin into autophagosomes to protect lens cells from cataract formation.

## Introduction

Autophagy is involved in physiological and pathological cellular processes including cell morphology, development, metabolism, inflammation, immunomodulation, cell growth, cell death, and cancer^1–11^. Autophagy is critical for maintaining normal cellular homeostasis, and cell function is compromised by autophagic dysregulation. Autophagy plays a housekeeping role in removing aggregated proteins and damaged organelles, such as mitochondria, endoplasmic reticulum and peroxisomes^12^. During autophagy, autophagosome formation is regulated by several autophagy-related (ATG) proteins^13,14^. Microtubule-associated protein 1 light chain 3 (LC3, mammalian homologue of yeast Atg8) and beclin 1 (mammalian homologue of yeast Atg6) are involved in the initial step of autophagy^15–17^. Increased beclin 1 expression and LC3-I/II conversion occur during autophagy in normal and cancer cells^15–17^. One of the best characterized substrates of autophagy is p62, which was initially identified as a signaling regulator that resides in the late endosome lysosome^18^. Impaired autophagy is accompanied by accumulation of p62, leading to the formation of large aggregates of p62 and ubiquitin^19^.

FYVE and coiled-coil [CC] domain containing 1 (FYCO1) was originally identified as a novel LC3-, Rab7-, and PI3P-interacting protein^20^. The LC3–FYCO1 interaction is mediated by an LC3-interacting region motif adjacent to the FYVE domain of FYCO1. FYCO1 localizes to the external but not the internal membrane of autophagosomes, and remains on the external surface of autolysosomes upon autophagosome/late endosome /lysosome fusion.

The lens is comprised of the lens capsule, lens epithelium and lens fibers. Autophagy plays a pivotal role in lens fiber cell maturation and the formation of the organelle free zone (OFZ). The lens epithelium at the anterior pole continually differentiates at the equatorial region to form fiber cells. Differentiating fiber cells lose their organelles to produce the OFZ, which is essential to lens transparency. Atg5 and FYCO1 play pivotal roles in maintenance of the OFZ and lens transparency^21^.

Cataract is the leading cause of vision dysfunction and blindness worldwide^22,23^. Cataractogenesis is a multifactorial process, and aggregation of misfolded crystallin proteins is a common feature of several cataract types^24^. Material that is surgically removed from cataracted lenses contains multiple species of lens proteins, many of which comprise high molecular weight protein aggregations that require denaturation by SDS, urea, or guanidinium hydrochloride for solubilization^24^. Human lens proteins are mainly comprised of α-, β- and γ-crystallins. α-crystallin is the major lens protein type and is comprised of two subunits A and B^25–27^. Cataract is thought to be a crystallin aggregation disease^28^.

During aging, the lens loses its transparency, leading to an age-related cataract^29^. In contrast, congenital cataracts appear within the first year of life due to genetic mutations. Mutations of more than 50 genes have been reported in congenital cataract^30^. Approximately 8.3–25% of congenital cataracts are hereditary^31–33^. Although FYCO1 is considered to be involved in human cataractogenesis, the exact mechanism is not completely understood. In the present study, we generated FYCO1 KO mice and identified cataract formation in these animals. We further elucidated the molecular mechanism of this phenotype, revealing that FYCO1 interacts with α-crystallin to protect lens cells from cataract formation.

## Results

### Analysis of FYCO1 tissue distribution and generation of FYCO1 KO mice

**Figure 1:**
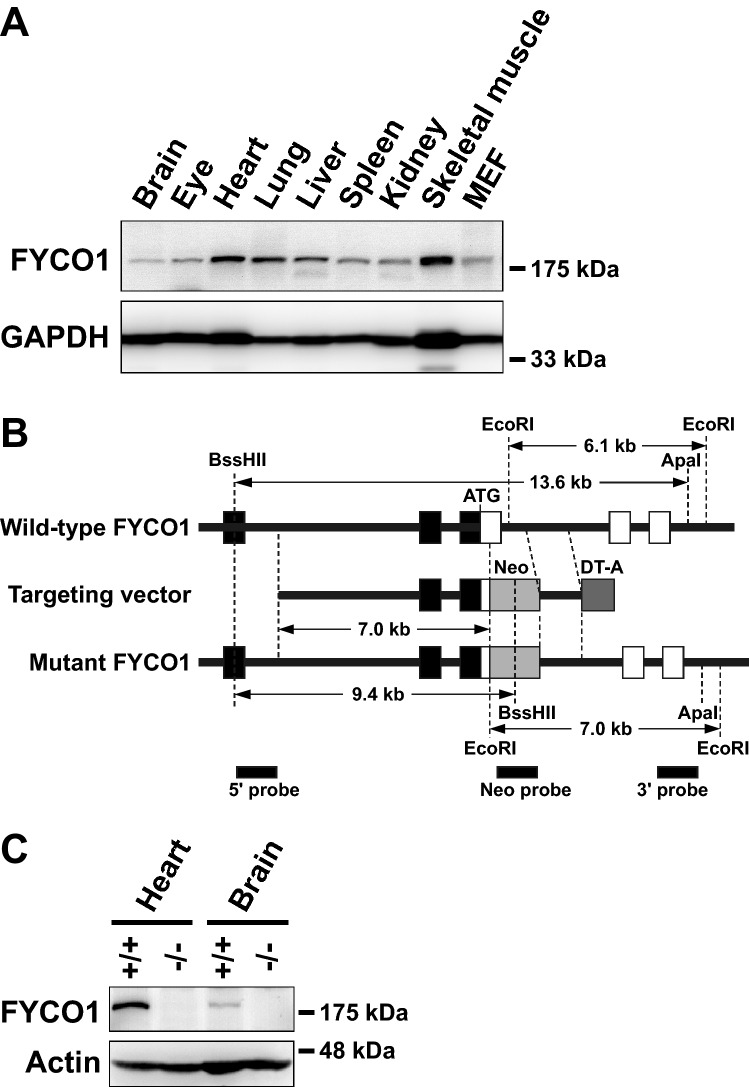
Analysis of FYCO1 tissue distribution and generation of FYCO1 knockout mice. (**A**) Extracts from various 4-week-old male C57BL/6J mouse tissues and MEFs (30 μg of protein) were subjected to western blot analysis with anti-FYCO1 antibody. Glyceraldehyde-3-phosphate dehydrogenase (GAPDH) was used as a loading control. This experiment was performed once using one mouse. (**B**) The mouse FYCO1 gene was disrupted by the insertion of a neomycin resistance gene cassette (Neo) in the first coding exon. Open and filled boxes represent coding and noncoding exons, respectively. The diphtheria toxin A gene cassette (DT-A) was inserted outside of the 3’ homologous region for negative selection. Restriction enzyme sites and probes used for Southern blot analysis are indicated. (**C**) Lysates from 4-week-old WT and KO mouse tissues (heart and brain) were subjected to western blot analysis with anti-FYCO1 antibody. This experiment was performed once using a mouse. (**D**) Lysates from 4-week-old WT and KO mouse tissues (eyes) were subjected to western blot analysis with anti-FYCO1 antibody. This experiment was performed once using a mouse.

We first determined which tissues and organs expressed FYCO1. Extracts from 4-week-old male C57BL/6J mouse tissues (brain, eye, heart, lung, liver, spleen, kidney, skeletal muscle and mesenchymal embryonal fibroblasts (MEFs) were subjected to western blot analysis with anti-FYCO1 antibody. FYCO1 was ubiquitously expressed in all tissues (Fig. 1A). We next generated FYCO1 KO mice to determine the function of FYCO1. To generate FYCO1 KO mice mouse FYCO1 gene was disrupted by the insertion of a neomycin resistance gene cassette (Neo) in the first coding exon. The open and filled boxes represent coding and noncoding exons, respectively (Fig. 1B). The diphtheria toxin A gene cassette (DT-A) was placed outside the 3’ homologous region for negative selection. Restriction enzyme sites and probes used for Southern blot analysis are indicated (Fig. 1B).

Extracts from 4-week-old wild-type and KO mouse tissues (heart, brain and eye) were subjected to western blot analysis with an anti-FYCO1 antibody. As expected, FYCO1 was not expressed in KO mouse tissues (heart and brain) (Fig. 1C) and eye (Fig. 1D).

We examined all organs and tissues of FYCO1 KO mice macroscopically and microscopically. We found lens opacities (cataracts). Other organs and tissues appeared to be normal (data not shown).

### Cataract formation in FYCO1 KO mice

**Figure 2:**
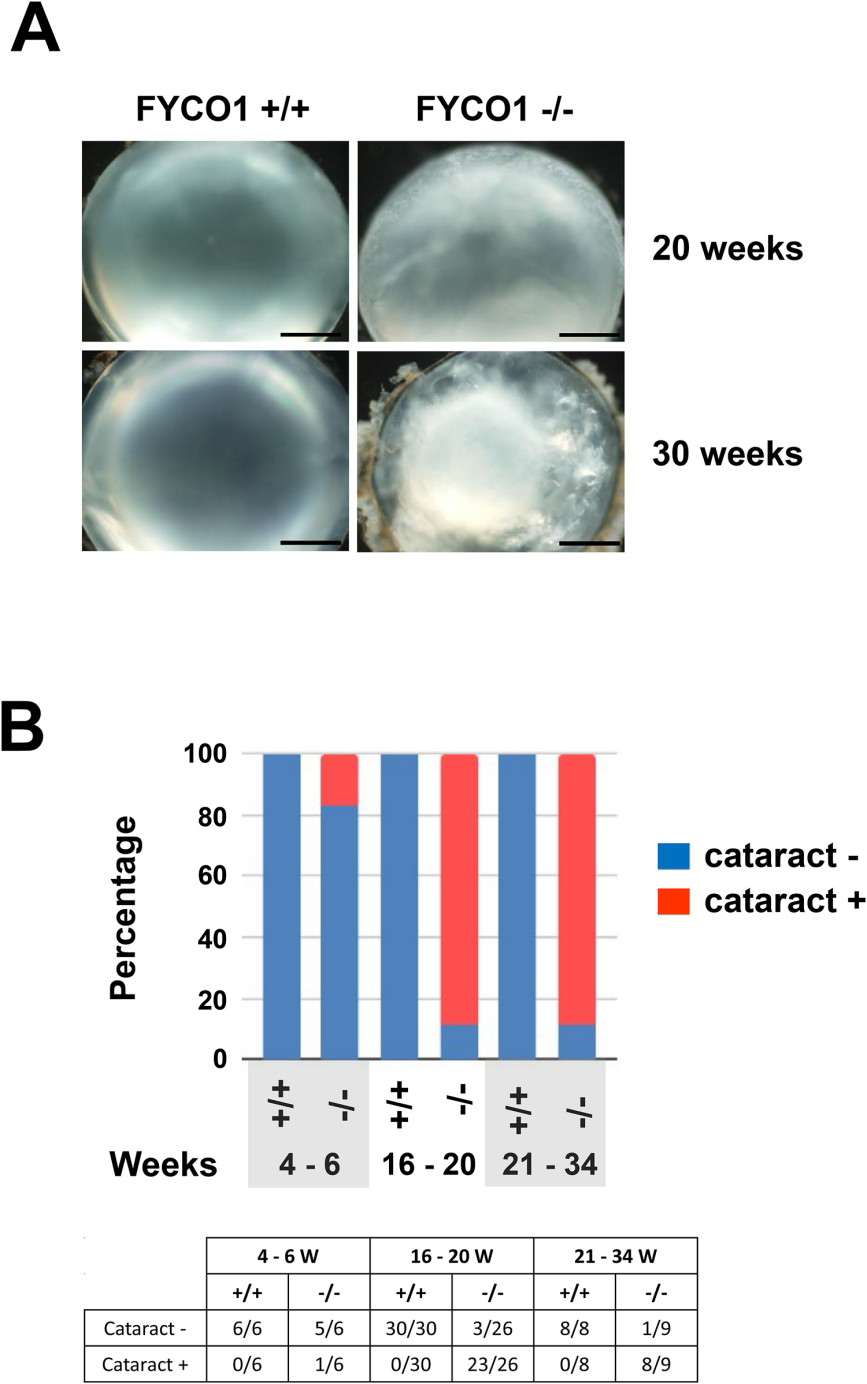
Cataract formation in FYCO1 KO mice. (**A**) Lenses from WT and FYCO1 KO mice at 20 and 30 weeks of age were dissected and photographed. Scale bars, 500 μm. This experiment was performed three times using a mouse per experiment. (**B**) The incidence of opacities (cataract) in WT and FYCO1 KO mice is shown. The table summarizes cataract incidence with age (4–6 weeks, 16–20 weeks and 21–34 weeks).

Subsequently, lenses from wild-type (+/+) and KO (−/−) mice at 20 and 30 weeks of age were analyzed. Lenses were dissected and photographed (Fig. 2A) (Scale bars, 500 μm). Lenses from wild-type (+/+) mice appeared normal (left panel). On the contrary, the lens from FYCO1 KO (−/−) mice clearly showed opacities (cataract). On the contrary, lenses from KO (−/−) mice exhibited gross opacities (cataract). Lenses from KO (−/−) mice at 20 and 30 weeks of age exhibited mild and severe cataracts, respectively (Fig. 2A, right panel).

The incidence of opacities (cataracts) in wild-type (+/+) and FYCO1 KO (−/−) mice is shown in Fig. 2B. The table summarizes incidence with age (4–6 weeks, 16–20 weeks, and 21–34 weeks). The incidence of opacities in FYCO1 KO mice increased with age. Contrastingly, the incidence of opacities in wild-type (+/+) mice remained at 0% until 34 weeks of age.

We determined the presence or absence of cataract formation after we observed lens under a microscope objectively. Although it is easy to distinguish normal from mild and moderate symptoms, it is difficult to classify severity. For example, it is difficult to distinguish mild from moderate due to subjectivity in observation and analysis. In Fig. 2, we classified the lens based on cataract (+) or cataract (−).

### Histological analysis of lens from FYCO1 knockout mouse

**Figure 3:**
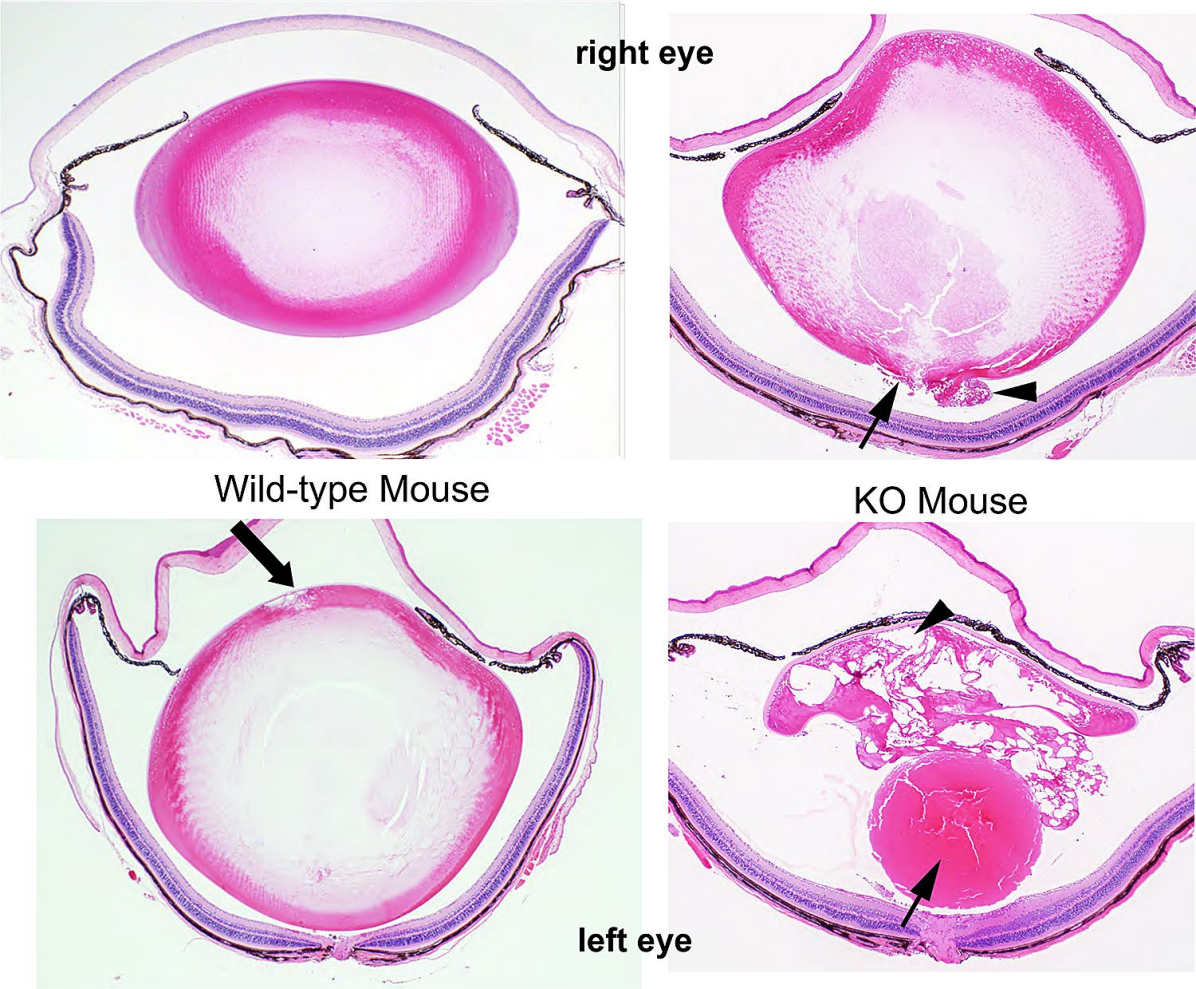
Histological analysis of lenses from wild-type and FYCO1 KO mice. Histological analysis of lenses from FYCO1 KO mice (right panel, 23 weeks) and wild-type mice (left panel, 23 weeks). Upper panel data are from right eyes and lower panel data are from left eyes. Serial Sects. (5 µm) were cut and stained with hematoxylin and eosin. This experiment was performed three times using one mouse per experiment.

Histological analysis of lenses from wild-type and KO mice was performed (Fig. 3).

Wild-type mice (left upper and lower panels) had almost normal lenses (left upper panel) except one lens with mild degeneration of lens fibers in the anterior pole of the lens (arrow, left lower panel). In KO mice (right panel), the lens capsule was ruptured in the posterior pole of the lens, and swollen lens fibers were prolapsed into the vitreous body with hypertrophied lens epithelium (right upper panel, arrow and arrowhead). In severe cases, the lens nucleus was also prolapsed to the vitreous body, and almost all lens fibers and epithelium were vacuolated and hypertrophied (right lower panel, arrow and arrowhead).

As shown in Fig. 3, we observed the phenotypical variability between the cataracts of the right and left eyes. The results suggest that there is a certain threshold for cataract formation. Crystallin aggregates and cataracts become more noticeable when the threshold is exceeded.

### Autophagy, p62 expression and α-crystallin solubility

**Figure 4:**
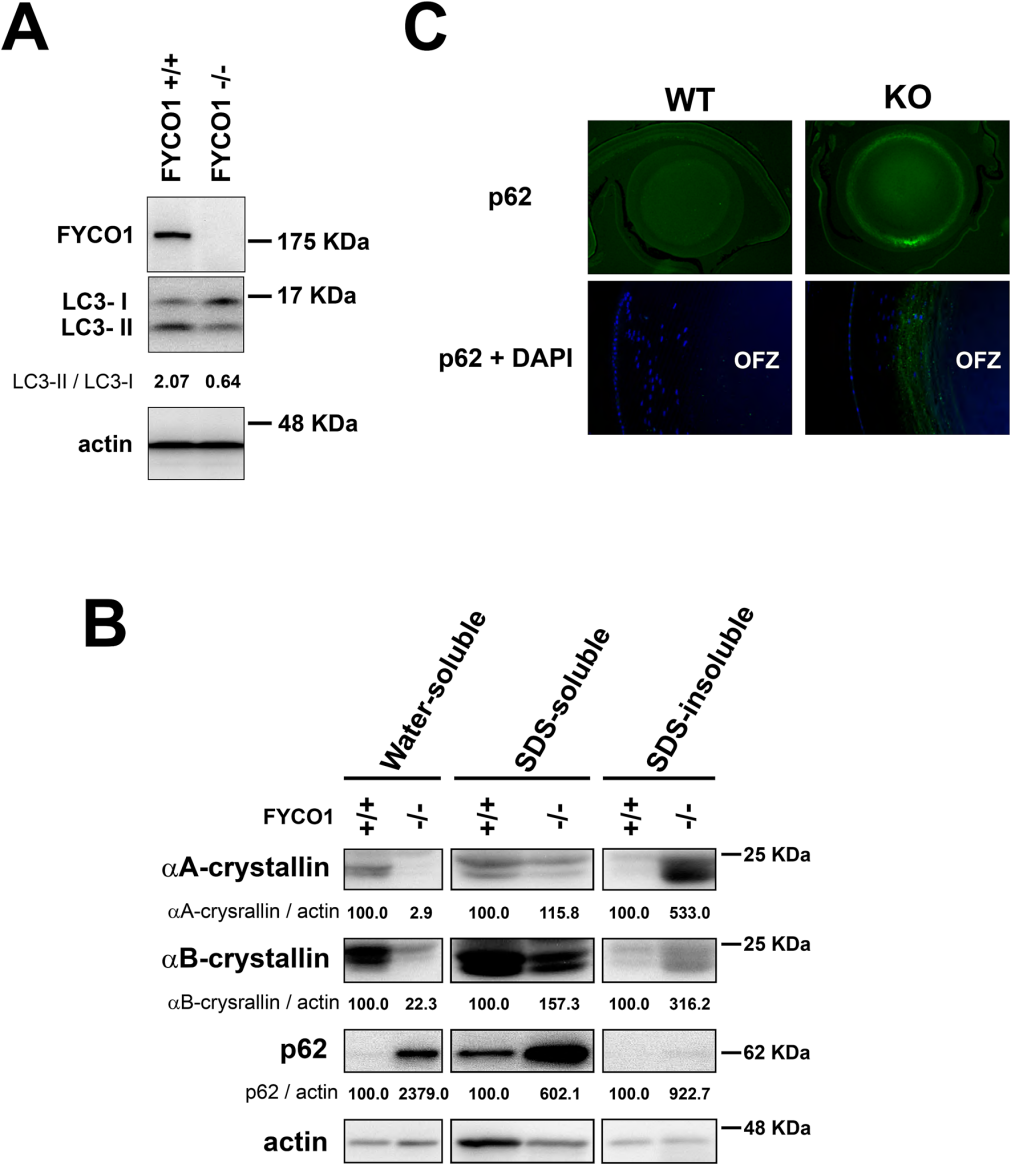
Impaired lens autophagy and decreased α-crystallin solubility in FYCO1 KO mice. (**A**) Conversion of LC3-I to LC3-II in lenses from WT (+/+) and KO (−/−) mice was analyzed by western blotting. Experiments were performed in duplicate with similar results, and a representative result is shown. This experiment was performed twice using a mouse per experiment. Both eyeballs from a 4-week-old mouse were used, and 50 µg protein was loaded in electrophoresis. (**B**) Analysis of αA-crystallin and αB-crystallin water solubility and water-insolubility (SDS-solubility and SDS-insolubility) in lenses from WT and KO mice. Protein levels of αA-crystallin and αB-crystallin were measured by western blot analysis. P62 levels were also assessed by western blot analysis. This experiment was performed twice using a mouse per experiment. Both lenses (right and left) were used and 10 µg protein was loaded in electrophoresis. (**C**) Immunohistochemical analysis of p62 expression. Lenses of WT and KO mice were stained with anti-p62 antibody. Mouse eyeballs were enucleated and immediately fixed using SUPER FIX (Kurabo). Paraffin sections were incubated with primary antibodies overnight at 4 °C, followed by secondary antibodies for 1 h. This experiment was performed twice using one mouse per experiment.

Because FYCO1 regulates autophagy, the conversion of LC3-I to LC3-II was quantified in lenses of wild-type (+/+) and KO (−/−) mice. The conversion of LC3-I to LC3-II was analyzed by western blotting. As shown in Fig. 4A, LC3-I/II conversion was active in wild mice (LC3-II/LC3-I = 2.07). Contrastingly, conversion of LC3-I to LC3-II was decreased in lenses of FYCO1 KO (−/−) mouse (LC3-II/LC3-I = 0.64; Fig. 4A). The results were reproducible. This suggested that autophagy activity was decreased in the eyes of the FYCO1 KO mouse.

α-crystallin, which is comprised of A and B subunits, is the major type of human lens protein. Because cataract is thought to be due to crystallin aggregation, αA-crystallin and αB-cystallin water solubility was analyzed in lenses from WT and KO mice. Water-soluble protein levels of αA-crystallin and αB-crystallin were assessed by western blotting. The protein levels of both water-soluble αA-crystallin and αB-crystallin were significantly decreased in lenses from KO (−/−) mice relative to WT (+/+) (2.9 vs. 100 and 22.3 vs. 100, respectively; Fig. 4B). The protein levels of both SDS-soluble αA-crystallin and αB-crystallin increased slightly in lenses from KO mice relative to WT (+/+) (115.8 vs. 100 and 157.3 vs. 100, respectively; Fig. 4B). The protein levels of both SDS-insoluble αA-crystallin and αB-crystallin were significantly increased in lenses from KO (−/−) mice relative to WT (+/+) (533.0 vs. 100 and 316.2 vs. 100, respectively; Fig. 4B). These data indicated that FYCO1 ablation decreased the abundance of water-soluble αA-crystallin and αB-crystallin proteins in the lens. Moreover, FYCO1 ablation significantly increased SDS-insoluble αA-crystallin and αB-crystallin proteins in the lens.

### p62 accumulation in FYCO1 KO mice

As impairment of autophagy is accompanied by p62 accumulation, p62 was next analyzed using western blotting. p62 was increased in the water-soluble and SDS-soluble fractions of eyes from FYCO1 KO (−/−) mice, relative to the WT (+/+) mice. p62 expression was also examined using immunohistochemistry. Ring-shaped p62 aggregation was observed in lenses from KO mice, while p62 expression was not observed in lenses from WT mice (Fig. 4C). Additionally, p62 expression was robust outside of the OFZ in lenses from KO mice (Fig. 4C). These data indicated that ablation of FYCO1 caused lens p62 accumulation, which was suggestive of impaired autophagy.

### Interaction between FYCO1 and αA/B-crystallin

**Figure 5:**
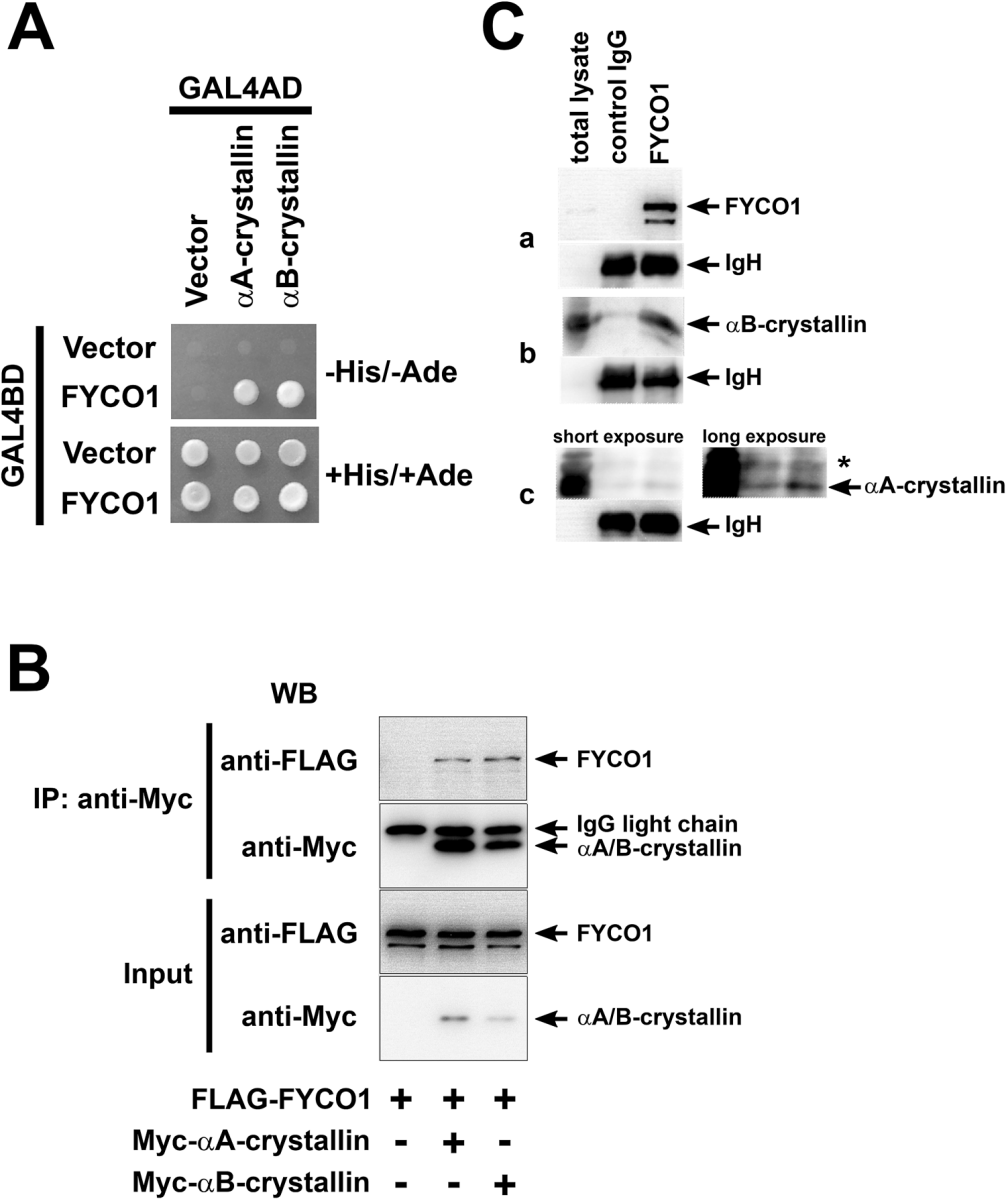
Interaction between FYCO1 and αA/B-crystallin. (**A**) Analysis of the interaction between FYCO1 and α-crystallin using the yeast two-hybrid system. Yeast Two-hybrid analysis using Matchmaker GAL4 Two-Hybrid System 3 (Clontech) was performed according to the manufacturer’s recommendations. This experiment was performed once. (**B**) Immunoprecipitation analysis of the interaction between FYCO1 and αA/B-crystallin. COS-7 cells were transfected with plasmids (0.1 µg pTarget-Myc-αA-crystallin, 0.1 µg pTarget-Myc-αB-crystallin and 1.5 µg pTarget-FLAG-FYCO1) as described in Materials and Methods, and cell extracts were subjected to immunoprecipitation (IP) with anti-Myc antibody and western blot (WB) analysis with the indicated antibodies. In the input lane, 2% of total reaction was loaded. + , present; -, absent. This experiment was performed twice. (**C**) Analysis of the interaction between FYCO1 and α-A/B-crystallin in wild (WT) mice eyeballs by immunoprecipitation and western blotting. FYCO1 associated with α-A/B crystallin in WT mouse eyeballs. Immunoprecipitates from WT mice eyeball lysate using FYCO1 antibody or control antibody were probed with α-A or α-B crystallin antibodies. Compared with immunoprecipitants using control antibody, those using FYCO1 antibody were detected with α-A and α-B crystallin. Blots were reprobed with anti-rabbit IgH antibody as a loading control. Asterisk indicates a nonspecific band. This experiment was performed twice using one mouse per experiment.

The yeast two-hybrid system was used to assess the hypothesis that FYCO1 interacted directly with α-crystallin. As shown in Fig. 5A, the results clearly showed the interaction between FYCO1 and αA-crystallin and FYCO1 and αB-crystallin.

To further confirm the interaction between FYCO1 and αA/B-crystallin, COS-7 cells were transfected as indicated, and cell extracts were subjected to immunoprecipitation (IP) with anti-Myc antibody and western blot (WB) analysis with the indicated antibodies. The interaction between FYCO1 and αA-crystallin and αB-crystallin was clearly confirmed (Fig. 5B).

We speculated that in the lens, FYCO1 is essential for clearance of degenerated crystallin. Therefore, we determined if FYCO1 associated with αA- and/or αB- crystallin in WT mouse eyeballs. When WT mouse eyeball lysates were immunoprecipitated with FYCO1 antibody, αB-crystallin (Fig. 5C-b) and αA-crystallin (Fig. 5C-c) were detected compared with lysates immunoprecipitated using control antibody (Fig. 5C-a). These data indicated that FYCO1 directly interacted with αA-crystallin and αB-crystallin in the lens.

### FYCO1 was not required for organelle degradation

**Figure 6:**
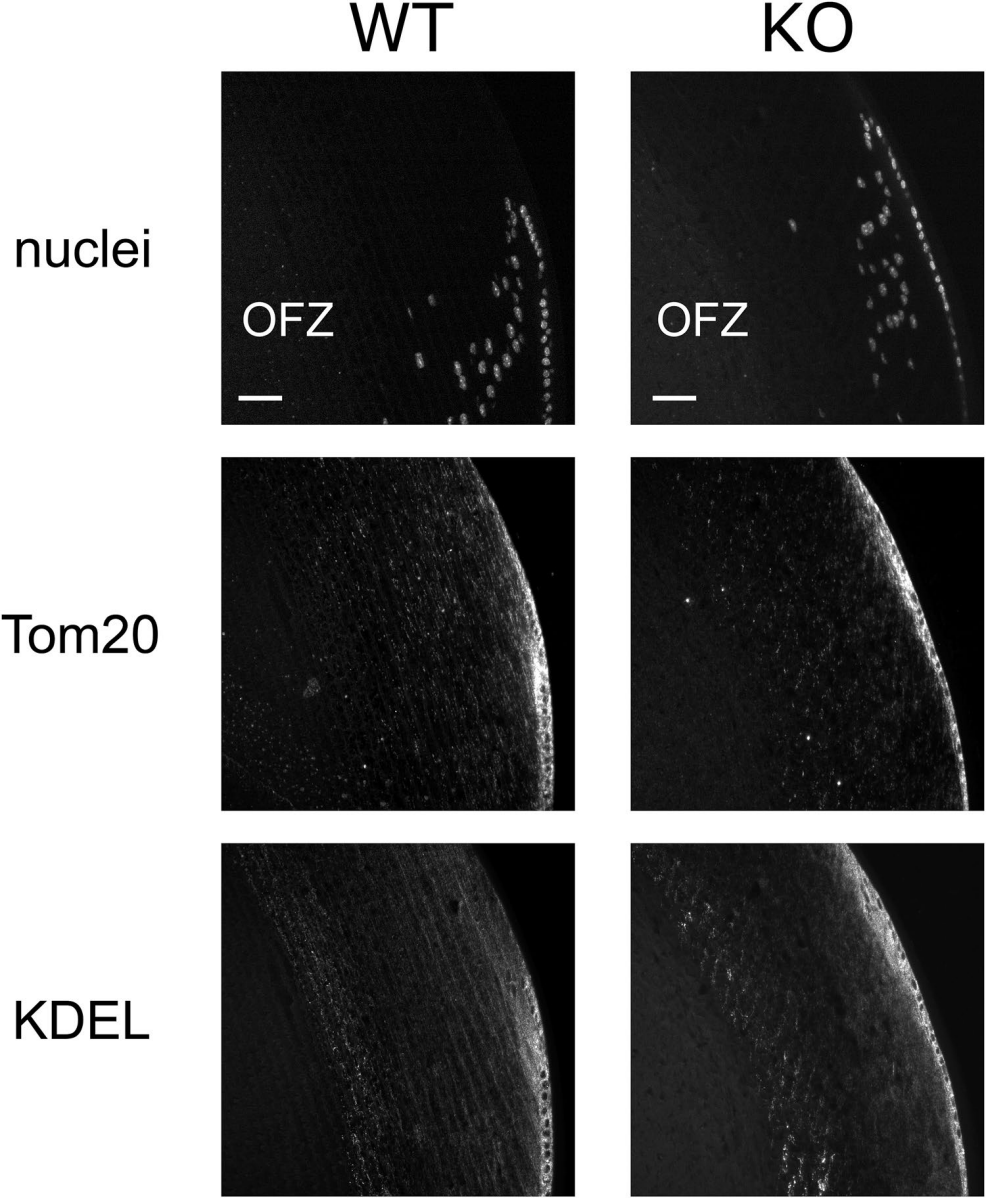
FYCO1 was not required for organelle degradation. The presence of nuclei, mitochondria, and ER in the cortical region and OFZ in lenses from 12-week-old WT and KO mice was evaluated using immunohistochemical staining with anti-KDEL antibody, anti-Tom20 antibody, and DAPI. Equatorial and cortical regions are shown. Scale bars, 40 μm. This experiment was performed twice using a mouse in each experiment.

We determined if FYCO1 was essential for organelle degradation. We stained paraffin sections of lenses with DAPI (nuclei staining dye), anti-Tom20 (mitochondrial marker protein), and anti-KDEL (ER marker protein GRP78). In lenses from WT and KO mice, nuclei, mitochondria, and ER were present in the cortical region but not in the OFZ (Fig. 6). These data suggested that FYCO1 was not required for organelle degradation in the OFZ.

### Proposed mechanism of action

**Figure 7:**
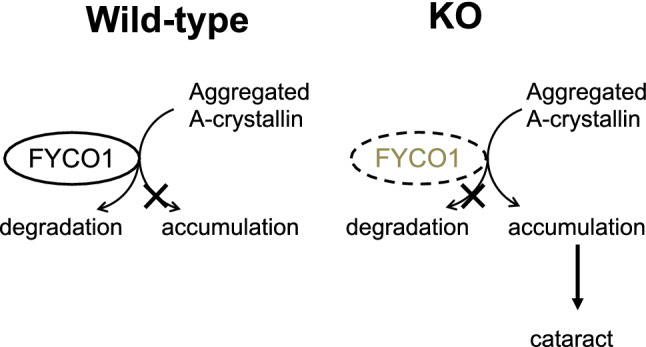
Proposed model of FYCO1 function in lens α-A/B-crystallin quality control. FYCO1 recruits damaged αA/αB-crystallin into autophagosomes and facilitates its degradation in autolysosomes to prevent cataract formation. In the case that wild-type mice FYCO1 positively controls autophagy, it will lead to degrade the aggregated αA-crystallin and αB-crystallin. On the other hand, due to decreased autophagy activity in the case of FYCO1 knockout mice, aggregated αA-crystallin and αB-crystallin accumulate, thereby leading to cataract formation.

The proposed model of FYCO1 function in lens protein quality control is illustrated (Fig. 7). FYCO1 recruited damaged αA/αB crystallin into autophagosomes and facilitated its degradation in autolysosomes to prevent cataract formation.

## Discussion

In the present study, we demonstrated that disruption of the FYCO1 gene in mice caused cataract formation. In eyes from FYCO1 KO mice, LC3 conversion decreased, and p62 accumulation increased, suggesting impaired autophagy. In addition, FYCO1 interacted directly with αA- and αB-crystallin, as demonstrated by yeast two-hybrid screening and immunoprecipitation analyses. In eyes from FYCO1 KO mice, water-soluble forms of αA- and αB-crystallin, the lens’s major components, were decreased. These findings suggested that FYCO1 recruited damaged α-crystallin into autophagosomes to protect lens cells from cataract formation. Based on these results, we have proposed a new mechanism for cataract formation, as illustrated in Fig. 7. FYCO1 recruits damaged α-crystallin (αA- and αB-crystallin) into autophagosomes and facilitates its degradation in autolysosome to prevent cataract formation (Fig. 7).

Cataract is the leading cause of blindness worldwide^22,23^, and is characterized by lens protein aggregation^24^. The human lens is comprised of three major types of proteins, α-, β- and γ-crystallins, which account for approximately 90% of all lens proteins^34,35^. The transparency of the lens depends on maintaining the tertiary structures and solubility of lens crystallin proteins. Homogenization of human lens in water or lysis buffer yields two fractions: a water-soluble fraction and a water-insoluble fraction^36,37^. About 50% of lens proteins from aged human eyes accumulate in the water-insoluble fraction^38,39^, and water-insoluble proteins are known to increase with aging^36,37^.

Yang et al. reported that lens epithelium soluble αA-crystallin and αB-crystallin are decreased in age-related and congenital cataracts^40^. These findings are consistent with those of the present study (Fig. 4B). Yang et al. did not assess the role of FYCO1. Mutations of more than 50 genes such as crystallin and HSF4, have been reported in congenital cataract^30^. Shiels and Hejtmancik suggested that when mutations in crystallins or other lens proteins are sufficient in and of themselves to cause rapid and direct protein aggregation, they usually result in congenital cataract formation^29^. However, these studies did not assess FYCO1.

Chen et al. reported that mutations in the genes encoding FYVE and coiled-coil domain containing 1 (FYCO1) are associated with human cataracts in 12 Pakistani families and one Arab Israeli family^41^. Nine different mutations were identified, including c.3755 delC (p.Ala1252AspfsX71), c.3858_3862dupGGAAT (p.Leu1288TrpfsX37), c.1045 C > T (p.Gln349X), c.2206C > T (p.Gln736X), c.2761C > T (p.Arg921X), c.2830C > T (p.Arg944X), c.3150 + 1 G > T, c.4127 T > C (p.Leu1376Pro), and c.1546C > T (p.Gln516X). Chen et al. discussed a new cellular and molecular entry point for understanding lens transparency and human cataracts. The frequency of FYCO1 mutations in the Pakistani population and identification of mutations that lead to cataract formation might be useful in genetic diagnosis and improved cataract treatment and prevention. Chen et al. further discussed that loss of FYCO1 function inhibits autophagosome transport from the perinuclear area to the periphery leading to vesicle accumulation and loss of transparency. However, they did not evaluate the interaction between FYCO1 and crystallin.

In summary, we demonstrated that disruption of the FYCO1 gene in mice resulted in cataract formation. In the eyes of FYCO1 KO mice, LC3 conversion was decreased. In addition, FYCO1 interacted with αA- and αB-crystallin. In eyes from FYCO1 KO mice, the soluble forms of αA- and αB-crystallin, the major lens protein components, were decreased. In addition, p62 accumulated in eyes from FYCO1 KO mice. These findings suggested that FYCO1 recruited damaged α-crystallin into the autophagosome to protect lens cells from cataract.

## Materials and methods

### Antibodies

The following antibodies were used for immunoprecipitation and western blot analysis: anti-human FYCO1 (Abnova, H00079443-A01), anti-LC3 (Nanotools, 5F10), anti-p62/SQSTM1 (Abnova, 2C11), anti-αA-crystallin (Santa Cruz Biotechnology, sc-22743), anti-αB-crystallin (Acris, AP20218PU-N), ant-FLAG (Sigma, M2), anti-Myc (Santa Cruz Biotechnology, 9E10), anti-GFP (MBL, 598), anti-actin (Santa Cruz Biotechnology, sc-1615), anti-Myc antibody (Santa Cruz Biotechnology, 9E10), anti-FLAG antibody (Sigma, M2) and anti-GAPDH (Santa Cruz Biotechnology, sc-32233). An antibody against mouse FYCO1 was generated by immunizing rabbits with a peptide containing amino acids 1258–1274 of mouse FYCO1, and was affinity-purified using the immunizing antigen immobilized on CNBr-activated Sepharose 4B beads (Amersham).

### Western blot analysis

Tissues and cells were homogenized in buffer A (20 mM Tris–HCl (pH7.4), 150 mM NaCl, 5 mM NaF, 1 mM Na_3_VO_4_, 500 μM EDTA, 200 μM AEBSF, 160 nM aprotinin, 10 μM bestatin, 3 μM E-64, 4 μM leupeptin, and 2 μM pepstatin A) containing 0.5% Triton X-100. Homogenates were centrifuged at 19,000 × g for 60 min, and the resulting supernatants were quantified using the Bradford protein assay (Bio-Rad). Equal amounts of protein were subjected to SDS-PAGE, and transferred to polyvinylidene difluoride (PVDF) membrane (Millipore). The membranes were probed with specific primary antibodies, followed by horseradish peroxidase-conjugated secondary antibodies. Chemiluminescence detection was performed with Immobilon Western Chemiluminescent HRP substrate (Millipore). Band density was quantified using the Image J.

### Fractionation of lens homogenates

Lenses from four-month-old wild-type and FYCO1 knockout mice were homogenized in buffer A without detergent. The homogenates were centrifuged at 19,000 × g for 10 min, and the resulting supernatants were designated as water-soluble fraction. The pellets were washed with buffer A, resuspended in buffer A containing 1% SDS, boiled at 95 °C for 3 min, and centrifuged at 19,000 × g for 10 min. The resulting supernatants were designated as SDS-soluble fractions. The pellets were resuspended in buffer A containing 1% SDS and 1.3 M 2-mercaptoethanol, boiled at 95 °C for 3 min, and centrifuged at 19,000 × g for 10 min. The resulting supernatants were designated as SDS-insoluble fraction.

### Generation of FYCO1-deficient embryonic stem cells

A FYCO1-targeting vector was constructed by inserting a neomycin resistance gene cassette into a site just downstream of the FYCO1 start codon, preserving 7.0 kb (5’) and 0.9 kb (3’) of the flanking homologous regions at the FYCO1 locus. The targeting vector was linearized with NotI and electroporated into B6N-S1Utr embryonic stem (ES) cells derived from C57BL/6J mice^42^. Embryonic stem cells were cultured on a feeder layer of mitotically inactivated mouse embryonic fibroblasts (MEF) using a co-culture method described previously^42^. Neomycin-resistant clones were selected with G418 and screened by PCR for homologous recombination at the FYCO1 locus (Primers 22 and 29 to amplify 962 bp mutant allele and primers 46 and 29 to amplify 753 bp wild type allele. The primer sequences were as follows: Primer 22 (5’-ACCAAATTAAGGGCCAGCTCATTCC-3’), Primer 29 (5’-GTGGTTGTGAGCTAAGACTGGTGCTG-3’), and Primer 46 (5’-ACATTTCTGTGGACTGTGAGAGTCCGAG-3’). Southern blotting analysis was performed using standard techniques (See Supplemental Fig. S1). In brief, 3 μg of DNA was digested with restriction enzymes (digestion with EcoRI or double-digestion with ApaI and BssHII), followed by electrophoretic separation on agarose gels. DNA was transferred to nylon membranes in 20 × SSC, and hybridized with DIG-labeled DNA probes. Nylon membranes were washed with 0.1 × SSC/0.1% SDS. DNA fragments were detected using alkaline phosphatase labelled anti-DIG antibodies (Roche) and CDP-Star chemiluminescent substrate (Roche). Ultimately, four homologous recombination ES clones were established.

### Animals

To generate FYCO1 deficient mice, targeted ES cells were aggregated with CD-1 embryos as described previously^42^. Chimeric males were crossed with C57BL/6J females to examine the germline transmission of the targeted allele. Heterozygous (FYCO1 ±) mice were then backcrossed to C57BL/6J mice for at least 5 generations. The resulting heterozygous mice were intercrossed to produce homozygous (FYCO1−/−) mutants. There were no phenotypical differences among the number of backcrosses.

Genotyping of mice was performed by PCR using the following primers: neo primer, 5’-ATTTTGAATGGAAGGATTGGAGCTACGG-3’, which is homologous to the neomycin resistance gene cassette; FYCO1 forward primer, 5’- TAAACAGGAAGGTGAAAAACTTGGAGG -3’, which lies just upstream of the 3’ homologous region in the targeting vector; and FYCO1 reverse primer, 5’-GTGGTTGTGAGCTAAGACTGGTGCTG-3’, which lies just downstream of the 3’ homologous region in the targeting vector. The 951-bp and 1095-bp fragments represented the wild-type and targeted alleles, respectively. C57BL/6J and CD-1 (ICR) mice were purchased from Charles River Laboratories Japan (Atsugi, Japan). In addition to eyes, other major organs of KO mice were examined macroscopically and histologically using light microscopy (data not shown).

### Approval of animal experiments

All animal experiments were conducted according to the institutional and national animal experimental guidelines. All experimental protocols were reviewed and approved by the Tokyo University Animal Care, Ethics and Experimentation Committee (Approval number 19-32).

### Plasmid construction

cDNAs for human FYCO1 and αB-crystallin were amplified by RT-PCR using human heart total RNA (Clontech), and subcloned into expression vectors. The identity of the PCR products was confirmed by sequencing analysis. Human αA-crystallin cDNA was kindly provided by Dr. N. Fujii (Kyoto University). FLAG-tagged human FYCO1, Myc-tagged human αA-crystallin, and Myc-tagged human αB-crystallin were subcloned into the pTARGET vector (Promega) for transient gene expression.

### Cell culture and plasmid transfection

COS-7 cells were cultured in Dulbecco's modified Eagle's medium (Sigma) supplemented with 10% fetal bovine serum. Purified plasmids (0.1 μg pTarget-Myc-αA-crystallin, 0.1 μg pTarget-Myc-αB-crystallin and 1.5 μg pTarget-FLAG-FYCO1) were transfected into COS-7 cells using Lipofectamine LTX (Invitrogen) according to the manufacturer’s protocols. After 72 h cells were harvested and lysed with 20 mM Tris–HCl (pH7.4), containing 150 mM NaCl, 5 mM NaF, 1 mM Na_3_VO_4_, 500 µM EDTA, 200 µM AEBSF, 160 nM aprotinin, 10 µM bestatin, 3 µM E-64, 4 µM leupeptin, 2 µM pepstatin A, and 0.5% Triton X-100. After centrifugation at 19,000 × g, for 60 min at 4℃, the protein concentration was determined using a Bradford assay (Bio-Rad Laboratories, Inc.).

Immunoprecipitation was performed using 100 µg protein lysates. Lysates were incubated with protein G-agarose beads at 4℃ overnight with agitation and subjected to immunoprecipitation with 2 μg anti-Myc antibody (Santa Cruz Biotechnology, 9E10). Protein G–agarose beads were washed four times in cold RIPA buffer. Sample buffer (50 mM Tris–HCl, pH6.8, 2% SDS, 6% 2-mercaptoethanol, 10% glycerol, 0.02% bromophenol blue) was added to Protein G–agarose beads and heated at 95 ℃ for 5 min. Samples were then analyzed by western blotting using the anti-Myc antibody (Santa Cruz Biotechnology, 9E10, 1:2,000) or anti-FLAG antibody (Sigma, M2, 1:8,000).

### Yeast two-hybrid analysis

Yeast two-hybrid analysis was performed using Matchmaker GAL4 Two-Hybrid System 3 (Clontech) according to the manufacturer’s recommendations. Briefly, the yeast reporter strain AH109 was co-transformed with plasmids encoding various protein regions fused to the GAL4 DNA-binding domain (GAL4BD) or GAL4 activation domain (GAL4AD). Protein–protein interactions were analyzed by growth of the transformants on minimum medium plates lacking histidine and adenine. To identify novel FYCO1-binding proteins, human brain and heart cDNA libraries were screened using various regions of human FYCO1 as bait. We performed yeast two-hybrid screening to identify FYCO1-binding proteins. In addition to αA-crystallin and αB-crystallin, LC3 and α-crystallin were also identified.

### Immunohistochemistry

Mouse eyes were dissected and fixed immediately using SUPER FIX (Kurabo). Paraffin sectioned were incubated with primary antibodies overnight at 4 °C, followed by incubation with secondary antibodies for 1 h. Sections were stained with 4′,6-diamino-2-phenylindole (DAPI, Dojindo).

### Immunoprecipitation

The following antibodies were purchased: rabbit polyclonal anti-FYCO1 (Bethyl Laboratories), mouse monoclonal anti-p62 (Abnova, 2C11), KDEL (Enzo Life Sciences, 10C3), rabbit polyclonal anti-Tom20 (Santa Cruz Biotechnology, FL-145), and normal rabbit IgG for immunohistochemistry (Santa Cruz Biotechnology). Alexa Fluor 488-conjugated anti-mouse IgG and Alexa Fluor 594-conjugated anti-rabbit IgG secondary antibodies were purchased from Molecular Probes.

Eyes were collected from C57 BL/6 mice and lysed in 1% Triton X-100 lysis buffer (1% Triton X-100, 10% glycerol, 150 mM NaCl, 2 mM EDTA) with proteinase inhibitor cocktail (Wako), using a glass homogenizer. Eye lysates (10 mg) were immunoprecipitated with rabbit anti-FYCO1 antibody (MBL) or with rabbit control antibody (Santa Cruz Biotechnology) with protein A sepharose beads (Sigma). Beads were washed four times with 1% Triton X-100 lysis buffer and resuspended in 2 × SDS-PAGE loading buffer. Immunoprecipitates were separated on SDS-PAGE and transferred to polyvinylidene difluoride (PVDF) membranes. Membranes were probed with rabbit anti-FYCO1 antibody and anti-αB crystallin (Enzo Life Science) antibodies followed by peroxidase-conjugated anti-rabbit IgG antibody (New England Biolabs) or anti-αA crystallin antibody (Abcam, 1H3.B8) and peroxidase-conjugated anti-mouse IgG antibody (New England Biolabs). Proteins were then visualized using ECL reagents.

### Pathological analysis

Eyes were harvested from WT and FYCO1 KO mice, and fixed in 4% paraformaldehyde in 0.1 M phosphate buffer (pH 7.4). After fixation for 24 h. The eyes were subjected to histological examination. The eyes were dehydrated in a sequential ethanol series (50–100%) by an automated processor and embedded in paraffin wax. Serial sections (5 µm) were cut and stained with hematoxylin and eosin.

This study was carried out in compliance with the ARRIVE guidelines (http://www.nc3rs.org.uk/page.asp?id=1357).

## Supplementary Information

Supplementary Figures.

Supplementary Legends.

## Abbreviations

FYCO1: FYVE and coiled-coil [CC] domain containing 1
LC3: Microtubule-associated protein 1 light chain 3
OFZ: Organelle free zone
DAPI: 4′,6-Diamino-2-phenylindole
MEF: Mouse embryonic fibroblasts
WT and KO mouse: Wild-type and knockout mouse
ATG: Autophagy-related
PVDF: Polyvinylidene difluoride
KO: Knockout
WT: Wild type

## References

[1] Parzych KR, Klionsky DJ (2014). An overview of autophagy: morphology, mechanism, and regulation. Antioxid. Redox Signal..

[2] Levy JMM, Towers CG, Thorburn A (2017). Targeting autophagy in cancer. Nat. Rev. Cancer.

[3] Kim KH, Lee MS (2014). Autophagy—A key player in cellular and body metabolism. Nat. Rev. Endocrinol..

[4] Shibutani ST, Saitoh T, Nowag H, Münz C, Yoshimori T (2015). Autophagy and autophagy-related proteins in the immune system. Nat. Immunol..

[5] Antonioli M, Di Rienzo M, Piacentini M, Fimia GM (2017). Emerging mechanisms in initiating and terminating autophagy. Trends Biochem. Sci..

[6] Menzies FM, Fleming A, Rubinsztein DC (2015). Compromised autophagy and neurodegenerative diseases. Nat. Rev. Neurosci..

[7] Boya P, Esteban-Martínez L, Serrano-Puebla A, Gómez-Sintes R, Villarejo-Zori B (2016). Autophagy in the eye: Development, degeneration, and aging. Prog. Retin Eye Res..

[8] Saha S, Panigrahi DP, Patil S, Bhutia SK (2018). Autophagy in health and disease: A comprehensive review. Biomed. Pharmacother..

[9] Choi AM, Ryter SW, Levine B (2013). Autophagy in human health and disease. N. Engl. J. Med..

[10] Boya P, Reggiori F, Codogno P (2013). Emerging regulation and functions of autophagy. Nat. Cell Biol..

[11] Mizushima N, Komatsu M (2011). Autophagy: renovation of cells and tissues. Cell.

[12] Glick D, Barth S, Macleod KF (2010). Autophagy: cellular and molecular mechanisms. J. Pathol..

[13] Xie Z, Klionsky DJ (2007). Autophagosome formation: core machinery and adaptations. Nat. Cell Biol..

[14] Tsukada M, Ohsumi Y (1993). Isolation and characterization of autophagy-defective mutants of *Saccharomyces cerevisiae*. FEBS Lett..

[15] Fujita N, Itoh T, Omori H, Fukuda M, Noda T, Yoshimori T (2008). The Atg16L complex specifies the site of LC3 lipidation for membrane biogenesis in autophagy. Mol. Biol. Cell.

[16] Ahn CH (2007). Expression of beclin-1, an autophagy-related protein, in gastric and colorectal cancers. APMIS.

[17] Wei S-H (2014). Disturbance of autophagy-lysosome signaling molecule expression in human gastric adenocarcinoma. Oncol. Lett..

[18] Moscat J, Diaz-Meco MT (2009). p62 at the crossroads of autophagy, apoptosis, and cancer. Cell.

[19] Komatsu M (2007). Homeostatic levels of p62 control cytoplasmic inclusion body formation in autophagy-deficient mice. Cell.

[20] Pankiv S (2010). FYCO1 is a Rab7 effector that binds to LC3 and PI3P to mediate microtubule plus end-directed vesicle transport. J. Cell Biol..

[21] Frost LS, Mitchell CH, Boesze-Battaglia K (2014). Autophagy in the Eye: implications for ocular cell health. Exp. Eye Res..

[22] Brian G, Taylor H (2001). Cataract blindness–challenges for the 21st century. Bull. World Health Organ..

[23] Liu YC, Wilkins M, Kim T, Malyugin B, Mehta JS (2017). Cataracts. Lancet.

[24] Moreau KL, King JA (2012). Protein misfolding and aggregation in cataract disease and prospects for prevention. Trends Mol. Med..

[25] Andley UP (2008). The lens epithelium: focus on the expression and function of the alpha-crystallin chaperones. Int. J. Biochem. Cell Biol..

[26] Andley UP (2007). Crystallins in the eye: function and pathology. Prog. Retin Eye Res..

[27] Wang X, Garcia CM, Shui YB, Beebe DC (2004). Expression and regulation of alpha-, beta-, and gamma-crystallins in mammalian lens epithelial cells. Invest. Ophthalmol. Vis. Sci..

[28] Chen XJ, Hu LD, Yao K, Yan YB (2018). Lanosterol and 25-hydroxycholesterol dissociate crystallin aggregates isolated from cataractous human lens via different mechanisms. Biochem. Biophys. Res. Commun..

[29] Shiels A, Hejtmancik JF (2017). Mutations and mechanisms in congenital and age-related cataracts. Exp. Eye Res..

[30] Behnam M (2016). A novel homozygous mutation in HSF4 causing autosomal recessive congenital cataract. J. Hum. Genet..

[31] Francois J (1982). Genetics of cataract. Ophthalmologica.

[32] Haargaard B, Wohlfahrt J, Rosenberg T, Fledelius HC, Melbye M (2005). Risk factors for idiopathic congenital/infantile cataract. Invest. Ophthalmol. Vis. Sci..

[33] Merin S, Merin S (1991). Inherited cataracts. Inherited Eye Diseases.

[34] Sharma KK, Santhoshkumar P (2009). Lens Aging: Effects of crystallins. Biochim. Biophys. Acta..

[35] Bloemendal H, de Jong W, Jaenicke R, Lubsen NH, Slingsby C, Tardieu A (2004). Ageing and vision: structure, stability and function of lens crystallins. Prog. Biophys. Mol. Biol..

[36] Bloemendal H, Bloemendal H (1981). The lens proteins. Molecular and Cellular Biology of the Eye Lens.

[37] Jaffe NS, Horwitz J, Podos SM, Yanoff M (1991). Lens and cataract. Text Book of Ophthalmology.

[38] Roy D, Spector A (1976). Absence of low-molecular-weight alpha crystallin in nuclear region of old human lenses. Proc. Natl. Acad. Sci. USA.

[39] McFall-Ngai MJ, Ding LL, Takemoto LJ, Horwitz J (1985). Spatial and temporal mapping of the age-related changes in human lens crystallins. Exp. Eye Res..

[40] Yang J, Zhou S, Guo M, Li Y, Gu J (2016). Different alpha crystallin expression in human age-related and congenital cataract lens epithelium. BMC Ophthalmol..

[41] Chen J (2011). Mutations in FYCO1 cause autosomal-recessive congenital *cataracts*. Am. J. Hum. Genet..

[42] Tanimoto Y (2008). Embryonic stem cells derived from C57BL/6J and C57BL/6N mice. Comp. Med..

