# Supplementary material for: Loss of FYCO1 leads to cataract formation
Source: Sci Rep. 2021 Jul 2;11:13771. doi: 10.1038/s41598-021-93110-1 (PMC8253827; doi:10.1038/s41598-021-93110-1)

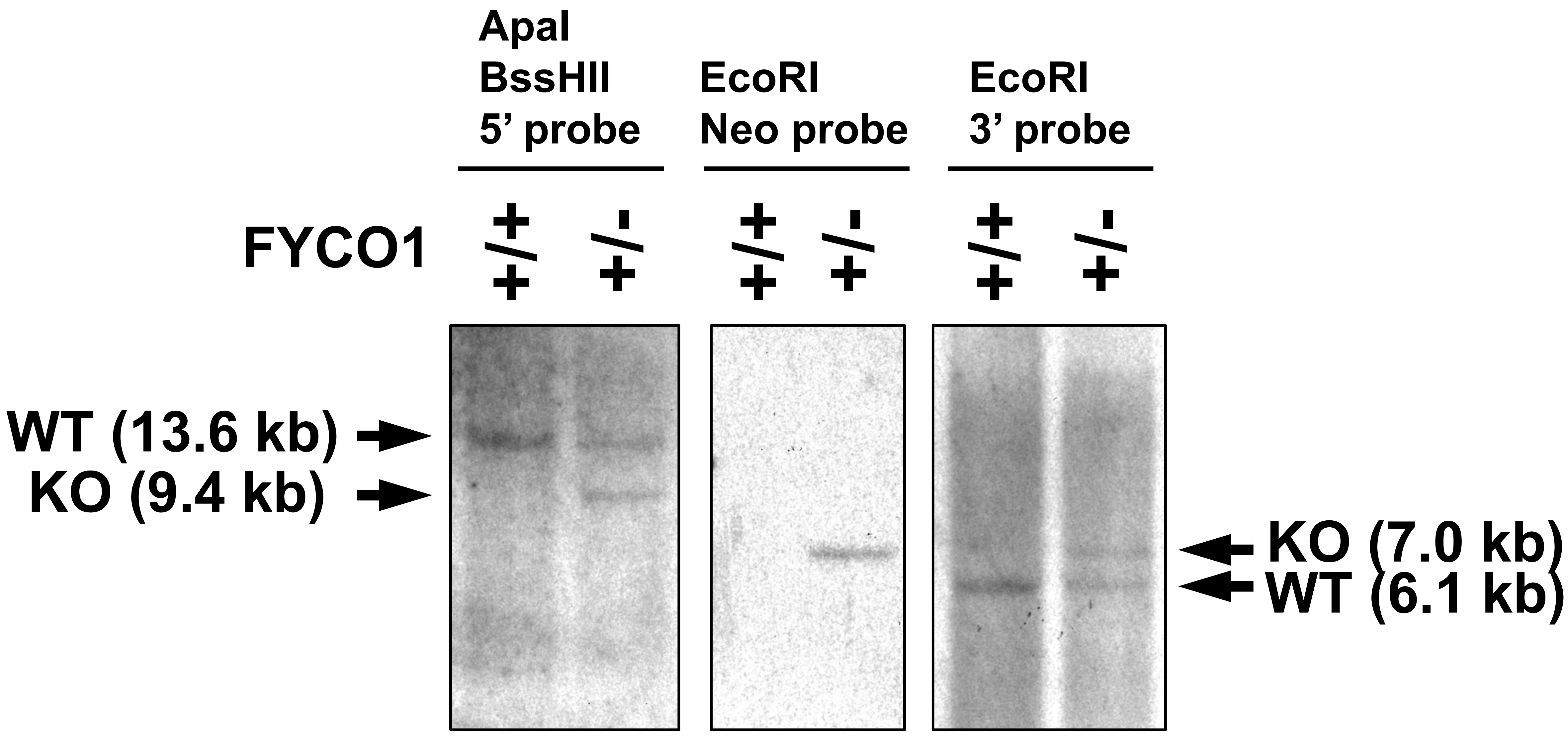

Supplementary Figure S1

Supplementary original whole blot Figure S1

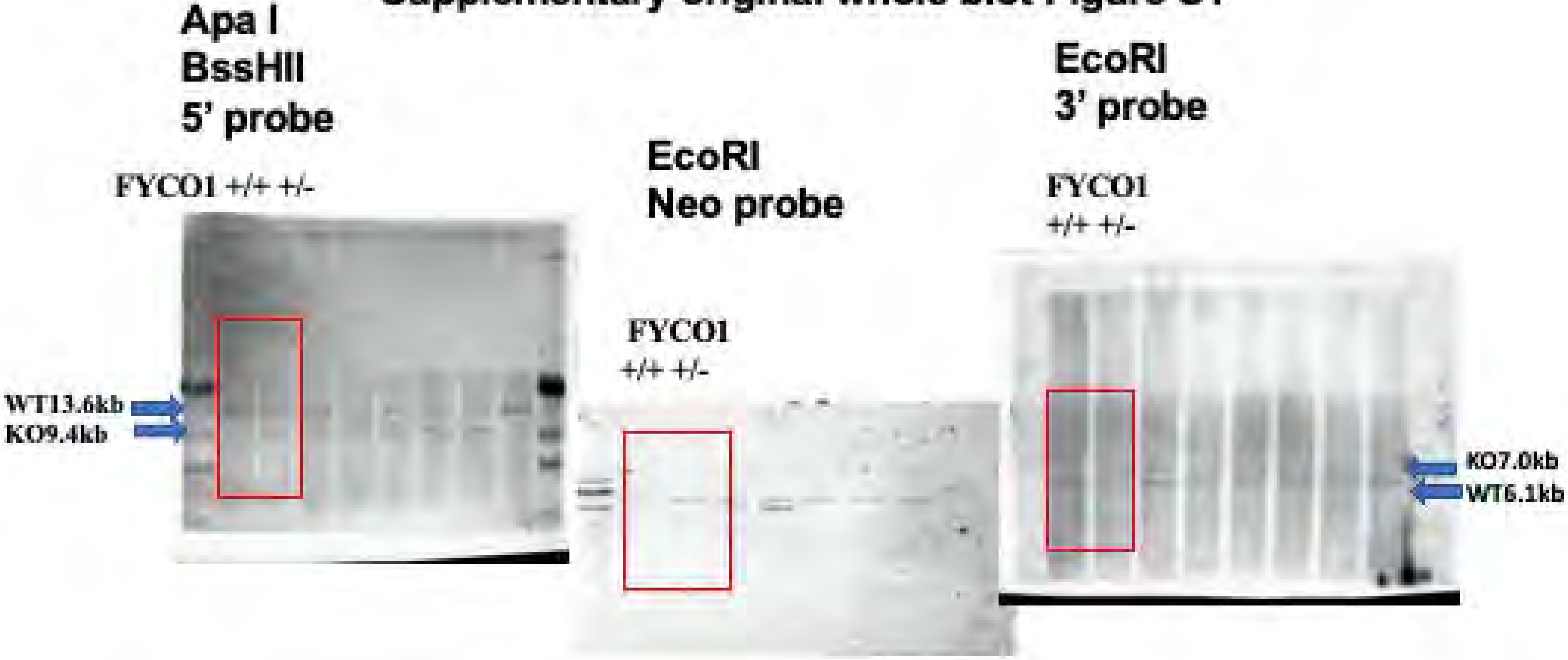

Supplementary Fig.1A
(original whole gel/blot)

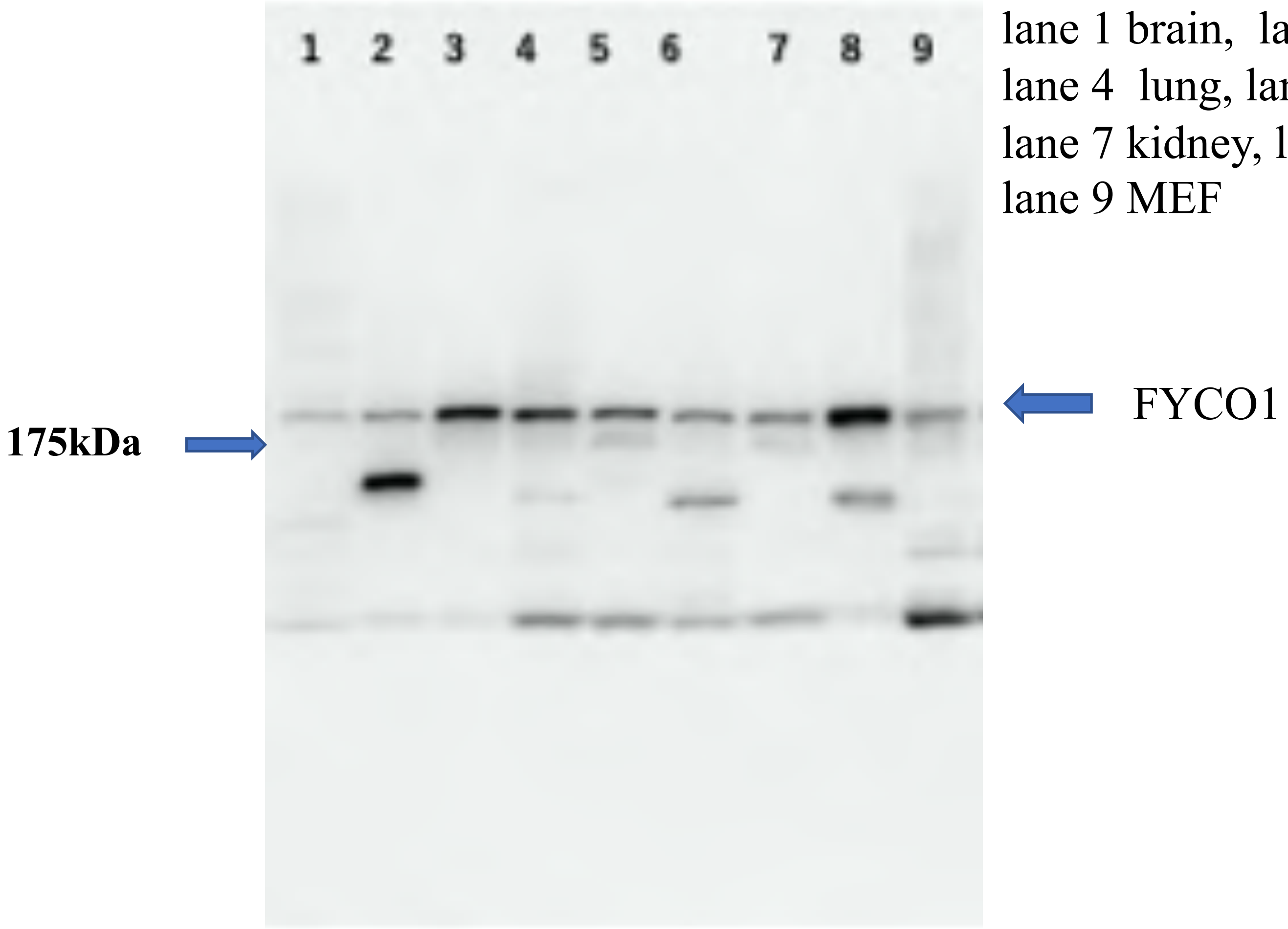

lane 1 brain, lane 2 eye, lane 3 heart
lane 4 lung, lane 5 liver, lane 6 spleen
lane 7 kidney, lane 8 skeletal muscle
lane 9 MEF

Supplementary Fig.1C
(Original whole gel/blot)

lane 1 heart +/+
lane 2 heart -/-
lane 3 brain +/+
lane 4 brain -/-

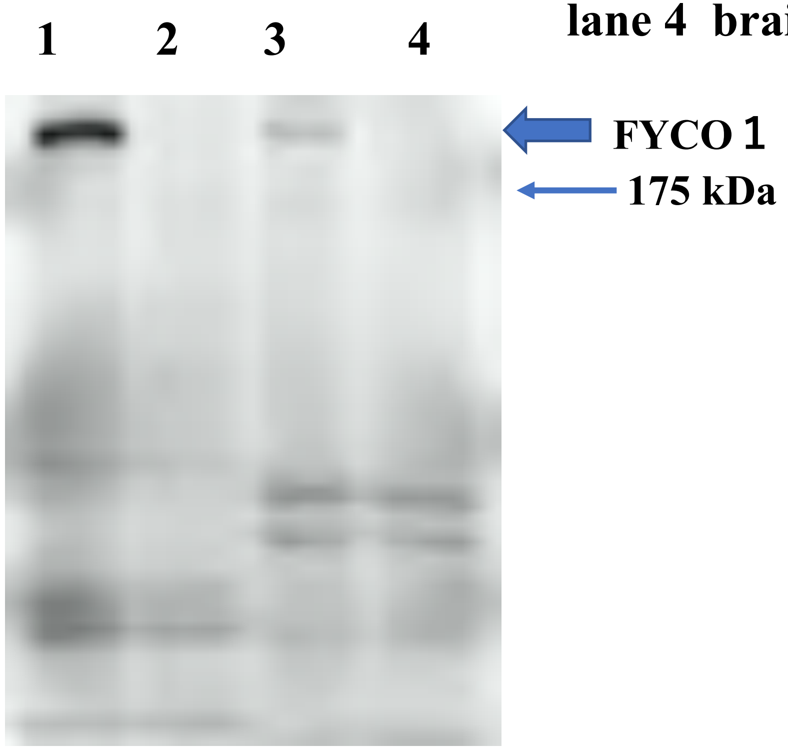

Supplementary Fig. 1 D
Original whole gel/blot)

Eyes

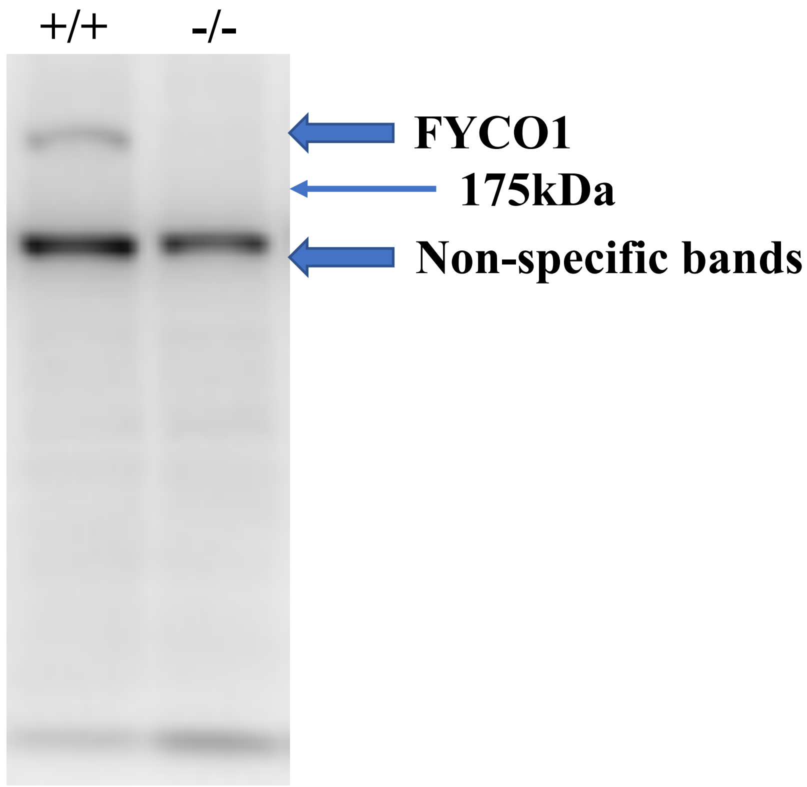

Supplementary Fig.4A
(original whole gel/blot)

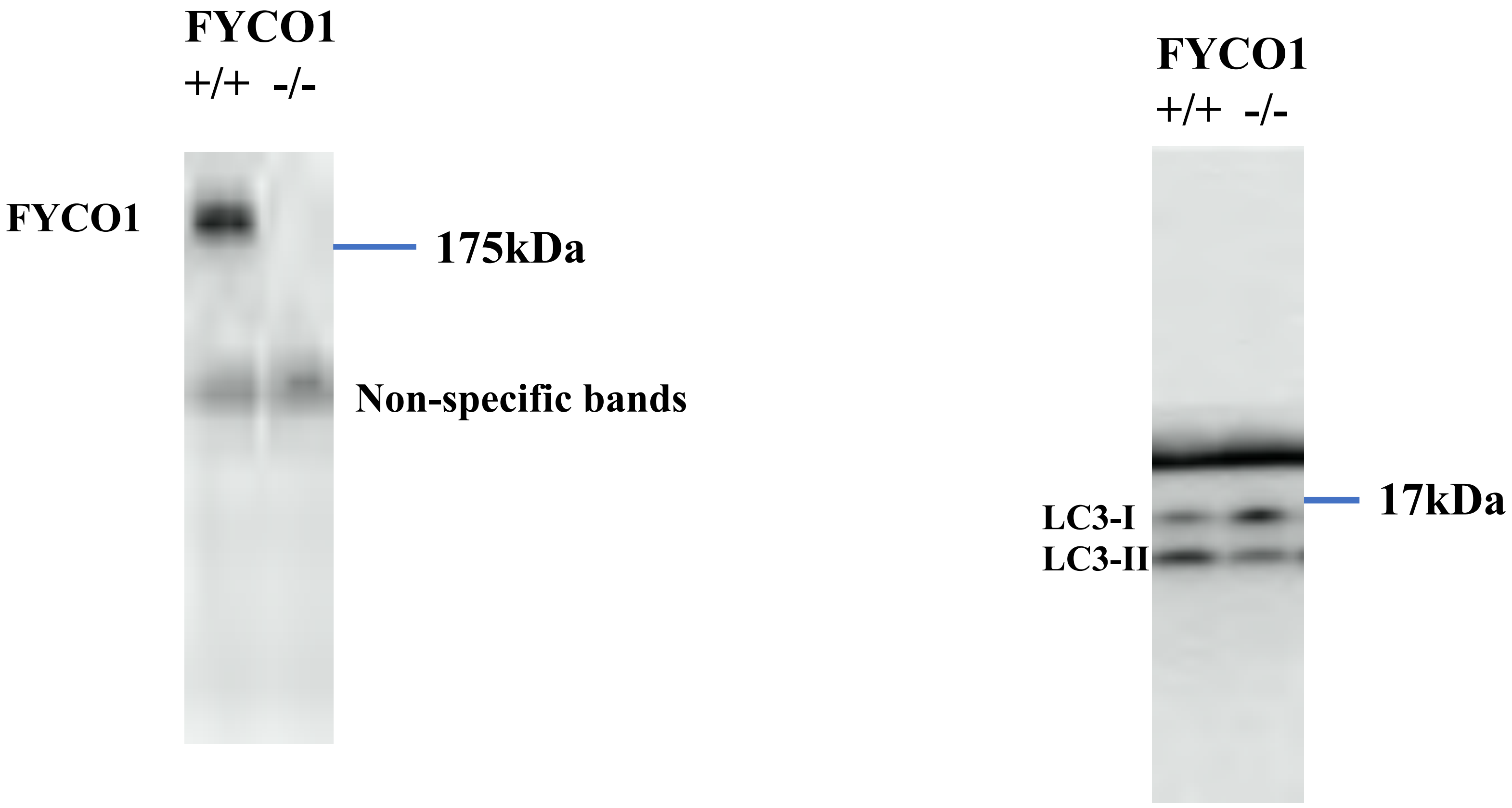

Supplementary Fig.4B (original whole gel/blot)

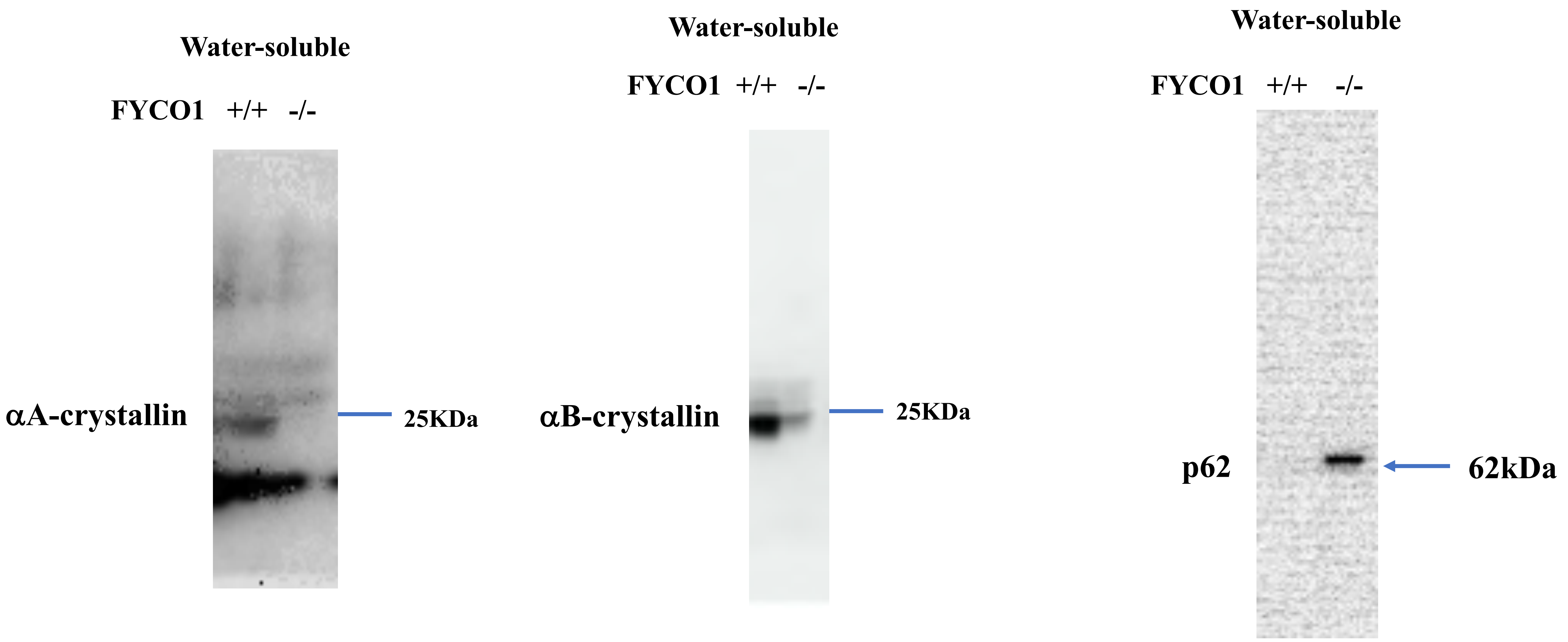

Supplementary Fig.4B (original whole gel/blot)

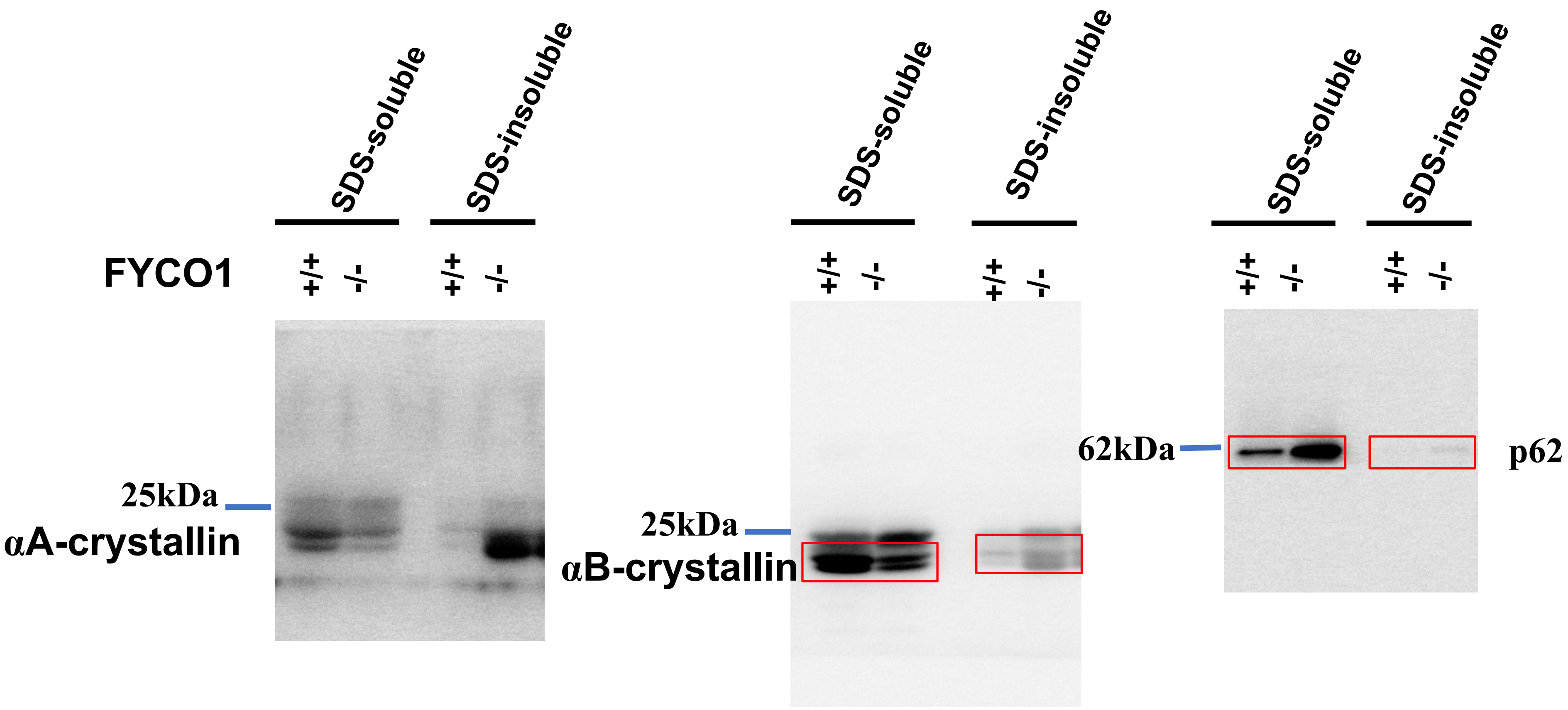

Supplementary Fig.5B original whole gels/blots

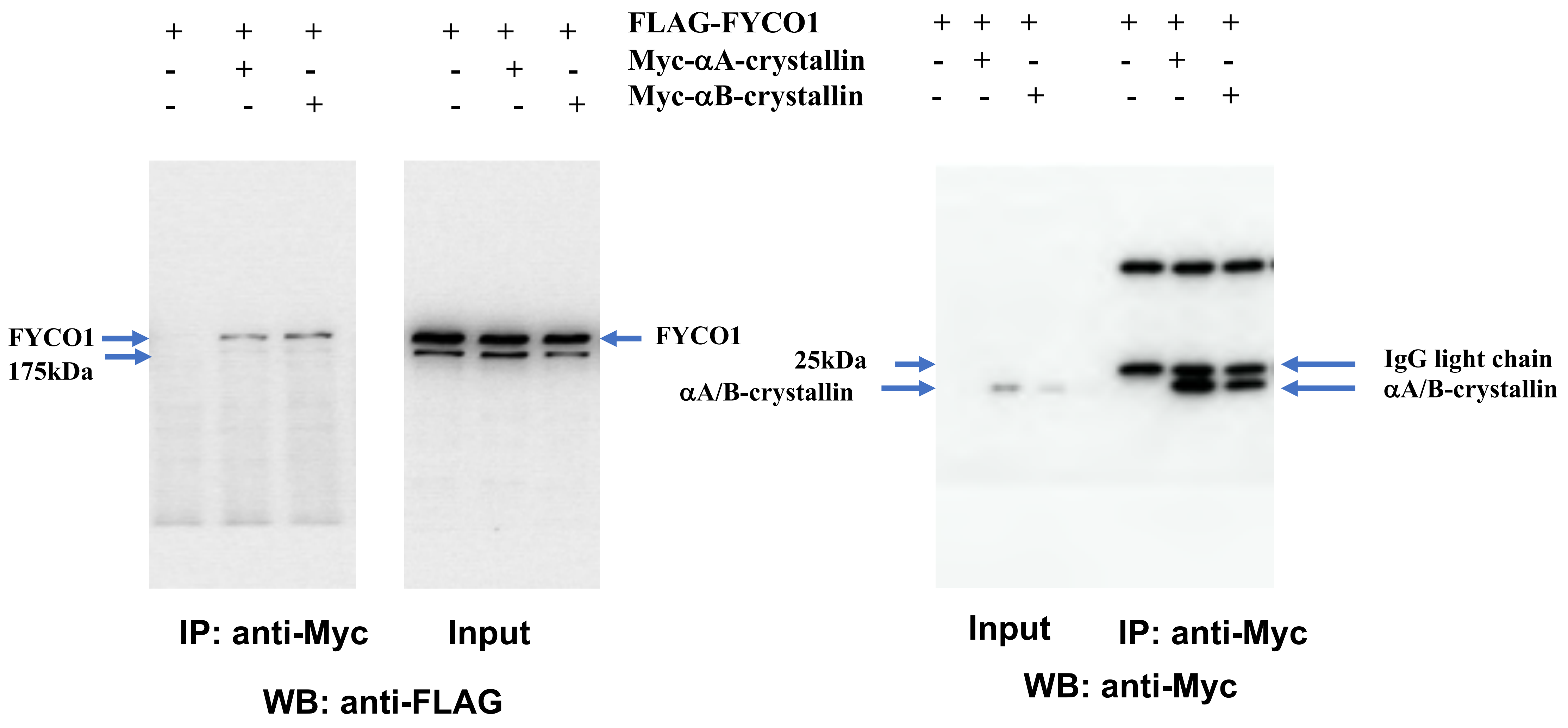

Supplementary Fig.5C-a

Lane 1 total eye lysate
Lane 2 control IgG
Lane 3 FYCO1

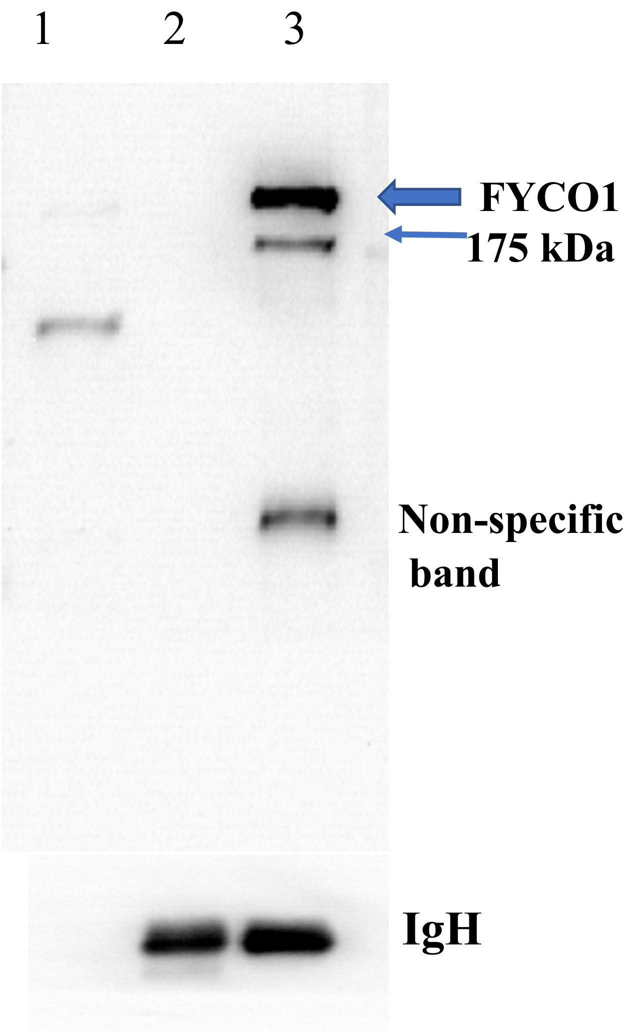

Supplementary Fig 5C-b

Lane 1 total lysate
Lane 2 control IgG
Lane 3 FYCO1

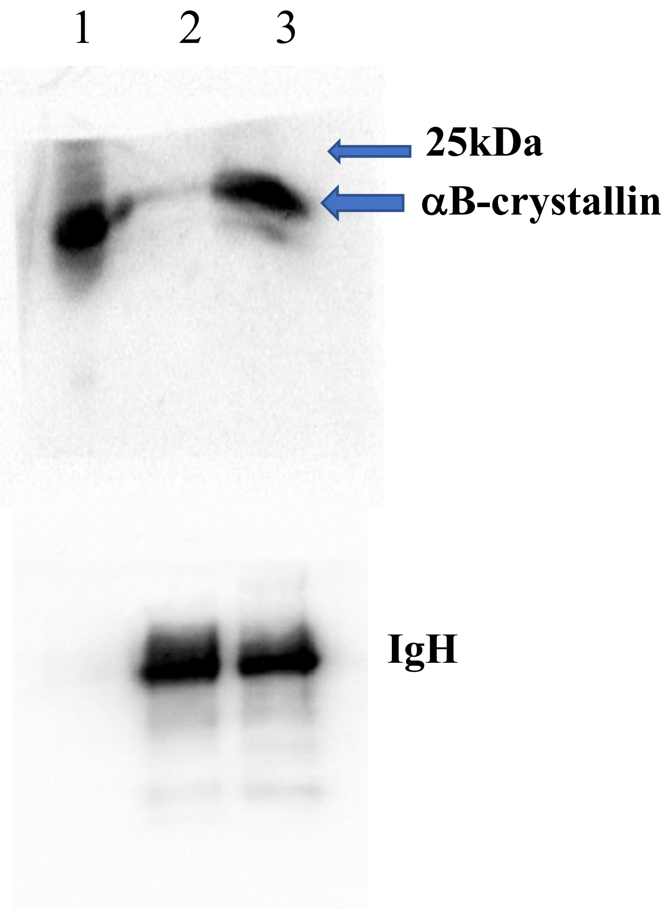

Supplementary Fig.5C-c

lane 1 total lysate
lane 2 control IgG
lane 3 FYCO1

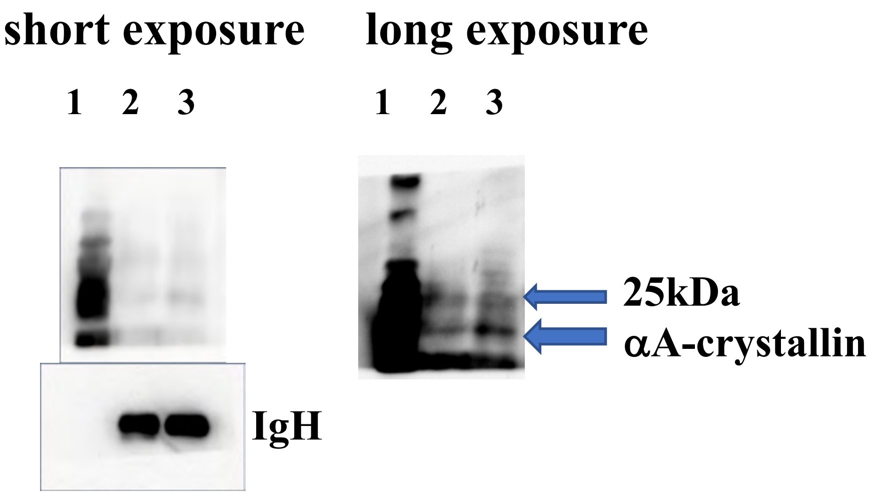

Supplement: Supplementary file 1 — Supplementary Figures. [file 41598_2021_93110_MOESM1_ESM.pdf]
