# Supplementary material for: Loss of FYCO1 leads to cataract formation
Source: Sci Rep. 2021 Jul 2;11:13771. doi: 10.1038/s41598-021-93110-1 (PMC8253827; doi:10.1038/s41598-021-93110-1)
Supplement: Supplementary file 2 — Supplementary Legends. [file 41598_2021_93110_MOESM2_ESM.docx]

**Loss of FYCO1 leads to cataract formation**

Kiyotoshi Satoh^1^, Yukitoshi Takemura^1^, Motohiko Satoh^1^, Kiyokazu Ozaki ^2^, Shunichiro Kubota^1,^*

^1^ Department of Life Sciences, Graduate School of Arts and Sciences, The University of Tokyo, 3-8-1 Komaba, Meguro-ku, Tokyo 153-8902, Japan

^2^Laboratory of Pathology, Setsunan University, 45-1 Nagaotohge-cho, Hirakata, Osaka,

Japan

* To whom correspondence should be addressed: Shunichiro Kubota,

E-mail: kubota.s787@gmail.com

Abbreviations:

FYCO1, FYVE and coiled-coil [CC] domain containing 1; LC3, Microtubule-associated protein 1 light chain 3; OFZ, organelle free zone; DAPI, 4’,6-diamino-2-phenylindole; MEF, mouse embryonic fibroblasts; WT and KO mouse, wild-type and knockout mouse; ATG, autophagy-related; PVDF, polyvinylidene difluoride; KO, knockout; WT, wild type;

**Legends for Supplementary original whole gels/blots**

**Supplementary Figure S1 (Southern blot analysis of genomic DNA from ES cells)**

Genomic DNA from ES cells was digested with restriction enzymes indicated above and subjected to Southern blot analysis with probes indicated in Fig. 1B.

**Supplementary Figure S1 (original whole blots)**

Genomic DNA from ES cells was digested with restriction enzymes indicated above

and subjected to Southern blot analysis with probes indicated in Fig. 1B. Whole blots

are shown. Blot surrounded by a square red frame were cropped and are shown as

Supplementary Figure S1.

**Supplementary Figure 1A (original whole blot)**

This figure is the original whole western blot for Figure 1A.

**Supplementary Fig.1C and Fig.1D (original whole blots)**

These blots are the original whole western blots for Figure 1C and 1D, respectively.

**Supplementary Fig.4A (original whole blots)**

These blots are the original whole western blots for Figure 4A.

**Supplementary Fig.4B (original whole blots)**

These blots are the original whole western blots for Figure 4B.

**Supplementary Fig.5B (original whole blots)**

These blots are the original whole IP-western blots for Figure 5B.

**Supplementary Fig.5C-a, 5C-b, and 5C-c (original whole blots)**

These blots are the original whole IP-western blots for Figure 5C.
